# Vascular lesions of the head and neck: an update on classification and imaging review

**DOI:** 10.1186/s13244-019-0818-3

**Published:** 2020-02-07

**Authors:** Akshaar N. Brahmbhatt, Kamila A. Skalski, Alok A. Bhatt

**Affiliations:** 1 Department of Imaging Sciences, University of Rochester – Strong Memorial hospital, 601 Elmwood Avenue, Rochester, NY 14642 USA; 2 Department of Radiology, Division of Neuroradiology, Mayo Clinic, 4500 San Pablo Road, Jacksonville, FL 32224 USA

**Keywords:** Vascular malformations, Vascular tumors, Head and neck, Arteriovenous malformations

## Abstract

Vascular lesions have a varied appearance and can commonly occur in the head and neck. A majority of these lesions are cutaneous and congenital; however, some may be acquired and malignant. The presentation and clinical history of patients presenting with head and neck lesions can be used to guide further imaging, which can provide important diagnostic and therapeutic considerations. This review discusses the revised International Society for the Study of Vascular Anomalies (ISSVA) classification system for vascular tumors and malformations, as well as explores the most common vascular anomalies including their clinical presentations and imaging findings.

## Key points

- The ISSVA has recently updated its classification of vascular anomalies.
- Vascular anomalies of the head and neck present unique diagnostic and therapeutic challenges.
- Age and clinical history/presentation are important factors in diagnosis.
- Vascular malformations and tumors may require multiple imaging modalities to fully characterize.

## Background

Vascular lesions within the head and neck have a broad pathological spectrum. These include a variety of tumors and malformations ranging from simple capillary irregularities to complex structures involving arteries, veins and lymphatics. There have been several classifications dating back to a histologically based version by Virchow in 1863. Since then, there have been many others based on the gross appearance, histological appearance, therapeutic treatment, and even flow rates. One of the most well-known is that of Mulliken and Glowacki, who published the first histology-based scheme, which described lesions in the pediatric population, dividing them into two major groups, tumors, and malformations [1, 2].

The most commonly used classification in use today comes from the International Society for the Study of Vascular Anomalies (ISSVA). It is a comprehensive scheme based on Mullikan and Glowacki’s seminal work. Overtime, advances in diagnostics, genetics, and treatments have led to several iterations of this classification, most recently in 2018. This updated version includes more lesions and reflects a more comprehensive understanding of underlying genetic causes. The ISSVA classification divides vascular lesions into two major categories: tumors (true proliferative neoplasms) and malformations (defects in morphogenesis).

The basic categories have remained the same. Vascular tumors are separated into benign, locally aggressive/borderline, and malignant (Table 1). The vascular malformations are divided into simple, combined, those of named vessels, and those associated with other anomalies (Table 2). It is important to understand these anomalies in the head and neck for two major reasons. Firstly, vascular anomalies most commonly occur in the head and neck. Secondly, these lesions have unique implications based on their anatomic location and involvement of major systems, e.g., visual, digestive, respiratory, etc. [2–4]. This review will focus on salient imaging findings and imaging-based prognostic considerations.

**Table 1:** Characteristics of benign, locally aggressive/borderline, and malignant vascular tumors

Vascular Tumors	Presentation	Imaging	Important Considerations
Benign			
Infantile Hemangioma	Present at birth.	Prominent serpiginous feeding vessel.	Delineate depth of lesion.
	Most commonly diagnosed in the first year of life. Rapid growth followed by slower growth and involution phases.	Iso- to intermediate T1 signal.	If lesion crosses multiple layers consider Kaposiform hemangioendothelioma.
		Hyperintense T2 signal. Increased T1 signal due to fibrofatty infiltration in the involuting phase and decreased contrast enhancement.	
Congenital Hemangioma	Usually present in the first year of life.	Intermediate T1 signal. High T2 signal.	Differentiated from infantile hemangiomas based on clinical course.
Tufted Angioma	Develops within the first five years of life with red or violaceous plaques.	Imaging rarely performed.	
Spindle Cell Hemangioma	Present as red or brown nodules.	Low T1 signal. High T2 signal lobulations.	Associated with Malfucci syndrome and Kaposiform hemangioendothelioma.
	May see associated lymphedema.	Often with phleboliths due to abnormal venous vasculature.	
Pyogenic Granuloma	Occur secondary to prior insult such as trauma or burns.	Isointense T1. Variable T2 signal.	
	Present with bleeding.		
Locally Aggressive/Borderline			
Kaposiform Hemangioendothelioma	Most common aggressive tumor.	Predominantly hyperintense T2 signal with aggressive features (ill defined margins, involvement of multiple tissue planes, stranding of the subcutaneous fat, and hemosiderin deposition due to prior hemorrhage).	
	Associated with thrombocytopenia and pain.	May involve adjacent bone.	
Hemangioendothelioma	Presents in adults as one or more slow growing nodules.	Usually evident on physical exam.	Distant metastasis rare.
Papillary Intralymphatic Angioendothelioma	Primary involves the skin and subcutaneous tissues.	Isointense T1 signal.	Distant metastasis rare.
		Heterogeneously increased T2 signal. Variable enhancement.	
		Often demonstrates local aggressive invasion.	
Malignant			
Angiosarcoma	Can occur at any age, mainly 7th and 8th decades of life.	Intermediate T1 signal intensity. High T2 heterogeneity.	Can metastasis to lung and bone via hematogenous spread.
	Most occur in the head and neck, particularly the scalp.	Avid, heterogeneous enhancement. Flow voids or high-flow serpentine loss of signal on T1 and T2 imaging in a soft tissue mass is characteristic.	
	Can occur secondary to chronic lymphedema and radiation.		
Epithelioid Hemangioma	Often incidental.	Can present as well marginated lucent lesions when involving osseous structures on radiography. Osseous lesions demonstrate T1 signal hyperintense to muscle. Heterogeneous high T2.	Potential for metastasis depending on grade.
	Typically presents between 30-50 years of age.

**Table 2:** Characteristics of vascular malformations

Vascular Malformations	Presentation	MR Imaging Characteristics	Additional helpful imaging characteristics
Capillary	Cutaneous lesions that often follow a dermal pattern.	Limited utility of imaging.	
		Can be used to exclude complicating components not obvious on physical exam.	
		Additional imaging can be performed if a syndrome is suspected.	
Macrocystic Lymphatic	Present at birth or by two years of age.	Complex multiloculated cystic mass with fluid-fluid levels. Cysts typically larger than > 2 cm. Enhancement of the walls and septa on post contrast imaging.	US: large cystic structures without internal vascularity.
	Soft large translucent non tender mass.		
Microcystic Lymphatic	Present at birth or by two years of age. Grow slowly in proportion to growing child.	Transpatial T1 hypointense T2 hyperintense lesion with multiple small cysts typically less than 2cm. No enhancement.	
	May appear as several small, raised sacs on the skin.		
Venous	Most common type of vascular malformation. Slow flow vascular malformation presents as a non pulsatile compressible soft tissue prominence or discrete mass typically with blueish-purple hue.	T2 hyperintense serpiginous tubules that enhance on delayed phase.	CT: serpiginous lesions that enhance on post contrast imaging. More sensitive for phlebolith identification.
	Often tracking along muscle groups or nerves. These are responsive to changes in flow i.e. Valsalva.	T2 hypointense focal phleboliths.	
Venolymphatic	Present with characteristics of both lymphatic and venous malformation.	Multiloculated cystic mass with fluid-fluid levels. Some enhancement on delayed phase images. Phleboliths can be present.	CT: serpiginous lesions, some areas can enhance on post contrast imaging. More sensitive for phlebolith identification.
	Classically soft nontender mass can have a blueish-purple hue.		
AVM	Can present with CHF, embolism, pain, or bleeding depending on location.	Serpiginous tangle of vessels with enhancement on post contrast imaging and early enhancement of the draining veins.	DSA remains the gold standard.
	High flow vascular malformation can present with pulsating lesion or thrill on examination. May feel warm on palpation.		Serpiginous tangle or mass of vessels with enhancement on post contrast imaging and early enhancement of the enlarged draining veins.
AVF	Symptoms depend on location and what vessels are involved. Can be asymptomatic and incidentally found.	Abnormal connection between an artery and vein, may present as abnormal dilation and fistulous tract on post contrast imaging.	DSA remains the gold standard and demonstrates direct communication of an artery with an abnormal early filling draining vein.
	Increased collateralization commonly seen.	Best seen on MRA which demonstrates abnormal early venous enhancement.	

Many of these vascular lesions have characteristic presentations and a comprehensive patient history plays a key role in establishing the correct diagnosis. Additionally, the clinical history can help recommend the imaging modality of choice and guide the clinician to the next most appropriate step. Most commonly, initial evaluation of most vascular lesions is often performed with ultrasound. This is followed by magnetic resonance imaging (MRI) for further characterization and to evaluate extent and structural involvement.

## Vascular Tumors

### Benign

#### Hemangiomas

Hemangiomas come in two forms infantile or congenital. Infantile hemangiomas are glucose transporter protein-1 (GLUT1) positive while congenital hemangiomas are not. Interestingly, GLUT-1 expression has been linked to poor prognosis in many neoplastic processes [5–7]. This over expression is thought to be due to increased need for glucose mediated by hypoxia. It is hypothesized that in utero episodes of hypoxia may lead to upregulation of GLUT-1 and angiogenic cytokines contributing to hemangioma formation [8, 9].

In addition to this genetic difference, they have several unique features that can help differentiate them. Infantile hemangiomas are usually diagnosed in the first year of life. They follow a pattern of rapid growth in the first few months followed by slower growth and involution phases that are slower and more variable. These lesions can be contrasted with congenital hemangiomas, which by definition are present at birth, and become symptomatic as they increase in size or in response to hormonal changes, infection and trauma.

#### Infantile Hemangiomas

**Fig. 1:**
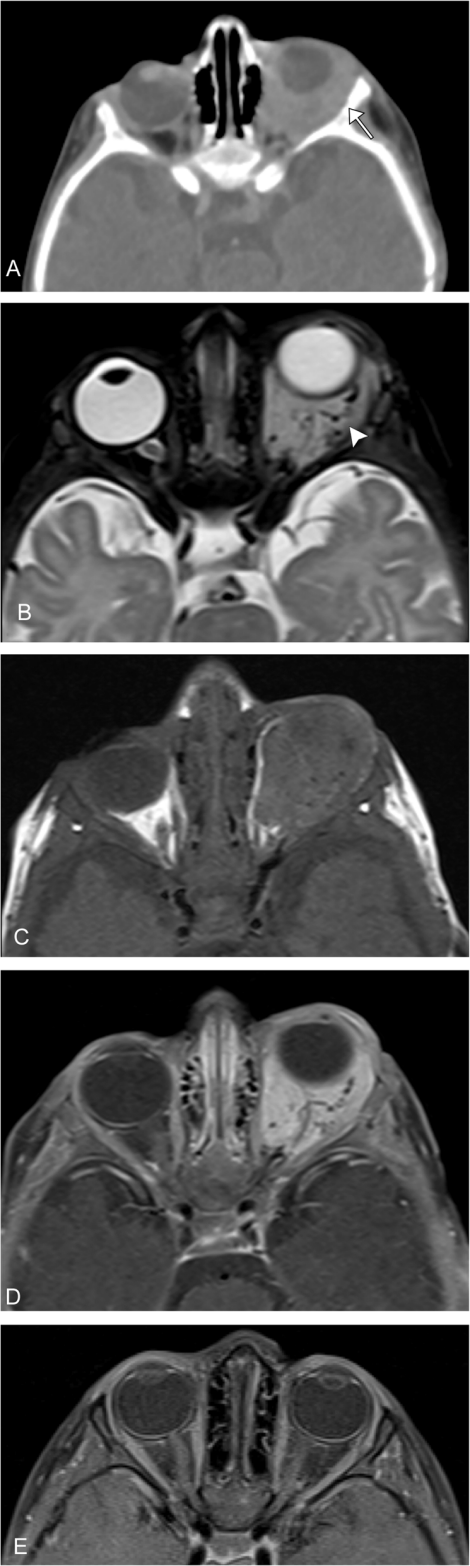
Infantile hemangioma. A 10-week-old full-term healthy infant presents with 1 week history of gradual swelling of the left eye. **a** Axial CT with contrast demonstrates an enhancing mass infiltrating the left intraorbital soft tissues, with resultant proptosis and smooth scalloping of the lateral orbital wall (arrow). **b** Axial T2 image demonstrates a mildly hyperintense mass (note: not as bright as cerebral spinal fluid) with prominent serpiginous vascular flow voids (arrowhead). **c** Axial T1 pre- and (**d**) post-contrast T1 fat suppressed images demonstrate avid enhancement of the lesion. **e** The patient was treated with propranol and post-contrast T1 fat suppressed imaging demonstrates resolution of the lesion

**Fig. 2:**
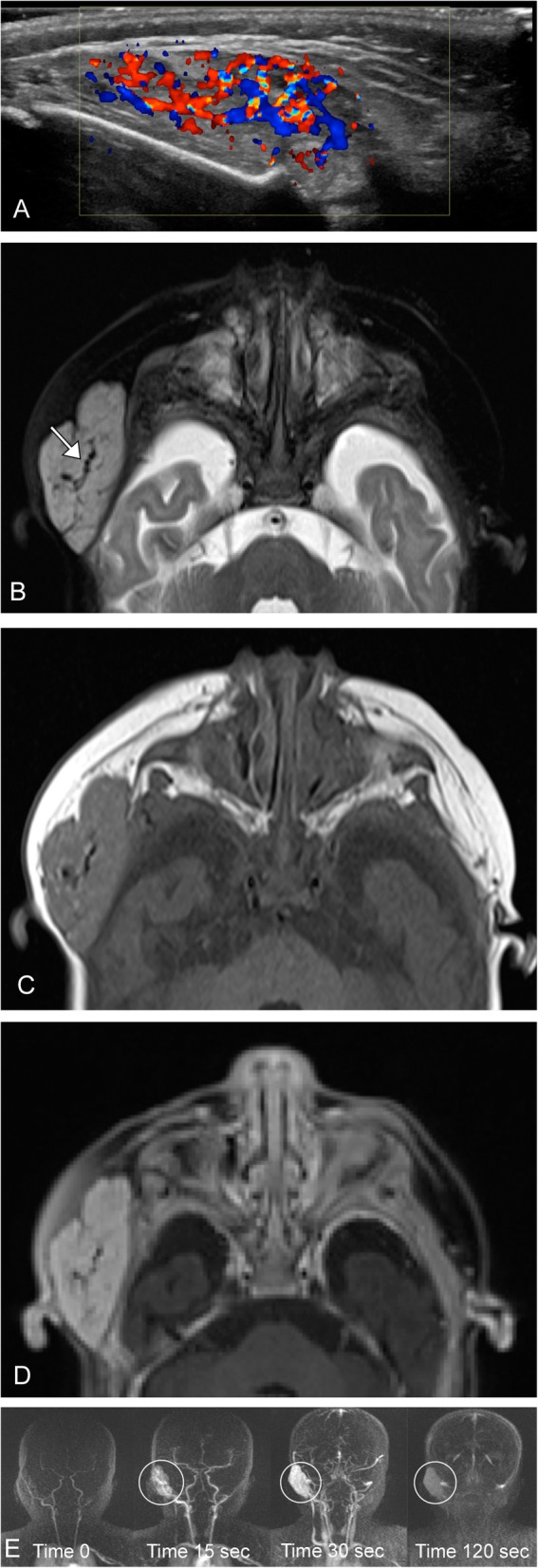
Infantile hemangioma. A-3 year-old female presents with a right pre-auricular face mass. **a** Ultrasound demonstrates a well circumscribed lesion at the area of concern with marked increased vascularity. **b** Axial T2 image demonstrates a mildly hyperintense mass (note: not as bright as cerebral spinal fluid) with lobulated borders and internal serpiginous vascular flow voids (arrow). **c** Axial T1 pre- and (**d**) post-contrast T1 fat suppressed images demonstrate avid enhancement of the lesion. **e** Time resolved contrast imaging demonstrates the classic wash in, followed by washout on delayed imaging (circled)

Infantile hemangiomas can be hyper or hypoechoic on ultrasound but demonstrate hypervascularity on color flow Doppler. These are best imaged on T1, T2, and post-contrast sequences when using MRI. Proliferating hemangiomas are iso to intermediate T1 and relatively hyperintense on T2 (however, not as hyperintense as cerebral spinal fluid), with enhancement after contrast administration. A serpiginous vessel with a prominent flow void denotes an arterial feeder, this along with lack of perilesional edema are important supporting findings. Dynamic contrast-enhanced imaging will demonstrate gradual, intense enhancement of the lesion, followed by gradual washout. In the involuting phase; these lesions demonstrate fibro-fatty infiltration with T1 hyper intense foci and decreasing contrast enhancement (Figs. 1 and 2).

These are further broken down based on their distribution into focal (single localized lesion, which is the most common), multifocal (several lesions), segmental (diffuse plaque like), and indeterminate [10]. In addition to the location, depth is used to characterize these lesions. The most common are superficial lesions limited to the skin. These are commonly dubbed “strawberry” lesions. Deep lesions occur under the skin and often appear bluish on gross examination. There are also mixed lesions with superficial and deep components. The last major subtype is reticular, abortive, minimal growth; these present as reticular or telangiectatic patches. They do not proliferate as readily and often involute faster than the other subtypes [11]. These hemangiomas are associated with posterior fossa malformations, arterial anomalies, cardiac abnormalities, and eye anomalies (PHACE syndrome).

It is important to contrast these lesions to Kaposiform hemangioendothelioma (KHE), which can also have a rapidly expanding phase, but is more infiltrative often crossing multiple soft tissue planes and often involving of the overlying skin. Most hemangiomas are clinically observed, but treatment maybe indicated if there is involvement of the airway, visual system, permanent disfigurement, or large lesions that may result in cardiac symptomatology. The mainstay of treatment is beta-blockers, namely propranolol [2].

#### Congenital hemangiomas

Congenital hemangiomas are similar to infantile hemangiomas in regards to their US and MRI findings. They are diagnosed retrospectively by their natural progression into rapidly involuting congenital hemangiomas (RICH), partially involuting congenital hemangiomas (PICH) and non-involuting capillary hemangioma (NICH) subtypes. RICH involute during the first year of life, in contrast to PICH and NICH. These lesions are associated with GNAQ/GNA11 genetic changes [10, 12]. Similar to infantile hemangiomas, most of these can be observed unless there is a complicating factor; the mainstay of treatment is beta blockers, but excision, laser therapy, or sclerosants can be used for residual lesions.

#### Tufted angioma

Tufted angioma is a rare vascular tumor that presents with red or violet plaques. Histologically, it is characterized by capillary vessels forming oval or rounded “tufts.” These develop usually within the first 5 years of life and can extend into many layers beyond the dermis. There is differing data on the location of these lesions but they tend to occur in the trunk and extremities in children. In adults, lesions are more likely to present in the head and neck, specifically the oral mucosa [13, 14]. It is important to biopsy these lesions to exclude a malignant neoplasm namely Kaposi sarcoma or angiosarcoma. Once pathological diagnosis is obtained, these lesions are usually observed; some of these lesions have known to regress. Treatment is usually symptomatic or cosmetic [15, 16]. Imaging is rarely performed on these lesions. Similar to KHE, these lesions have also been associated with GNA14.

Spindle cell hemangioma can present as a red–brown nodule ranging from a few millimeter to a few centimeter in diameter. They can present as a solitary lesion or cluster of several lesions. These lesions can be painful and have been associated with lymphedema, Mafucci syndrome, and Klippel–Trenaunay syndrome. These are mostly superficial lesions limited to the dermal and subcutaneous tissues. On MRI, they have low T1 signal and lobulated high T2 signal, often with phleboliths due to abnormal venous vessels. The most common treatment is surgical excision. While imaging is of limited utility, in select cases it can be helpful prior to treatment to characterize extent and when extensive reconstruction is expected [17, 18].

**Fig. 3:**
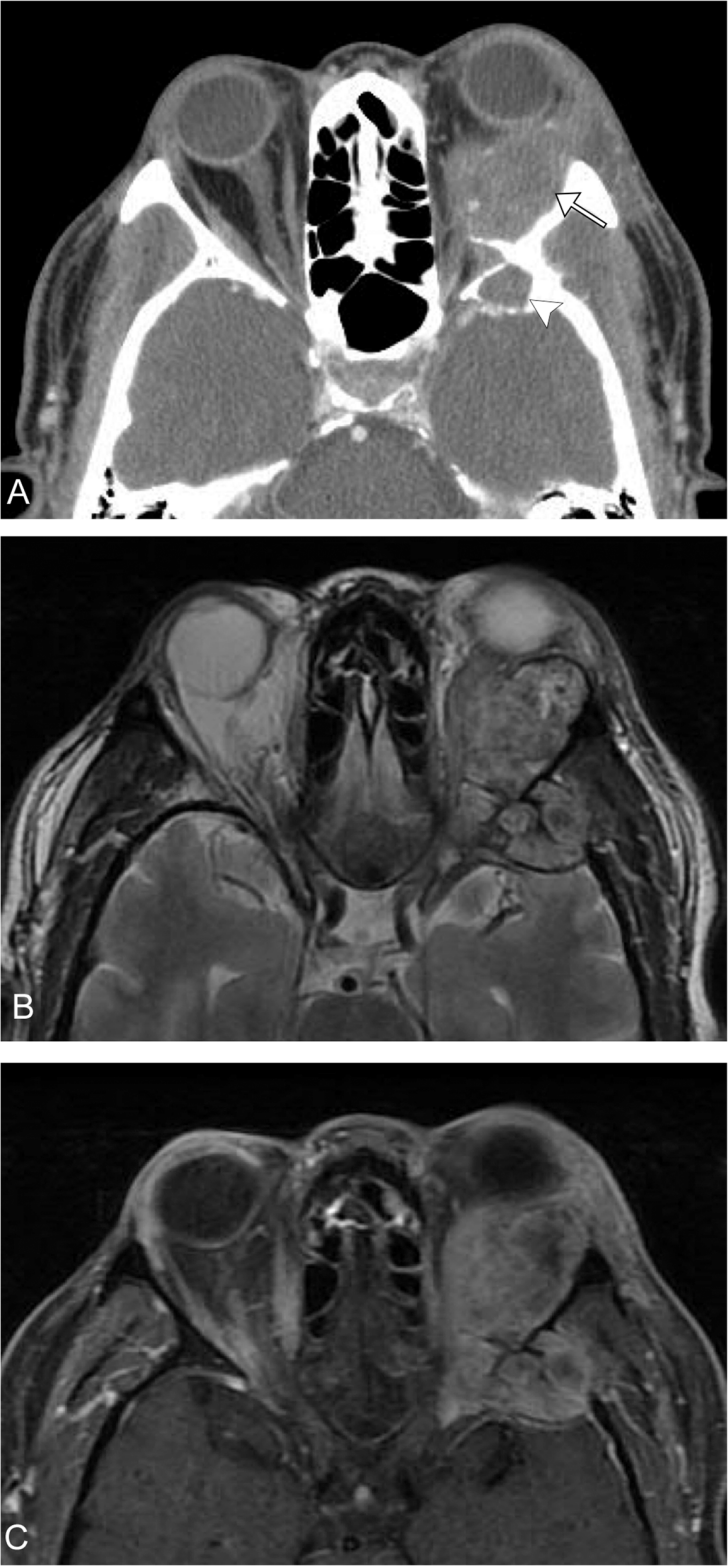
Epithelioid hemangioma. A 55-year-old male, with gradual left eye proptosis. **a** Axial CT demonstrates a multinodular mass centered along the lateral margin of the left orbit (arrow), also involving the left sphenoid wing (arrowhead). **b** Axial T2 image demonstrates the mass to be heterogeneous in appearance. **c** Axial T1 post-contrast fat-suppressed image demonstrates heterogeneous enhancement. These imaging features are non-specific. The patient underwent biopsy, which revealed epithelioid hemangioma

Epithelioid hemangiomas, previously named angiolymphoid hyperplasia with eosinophilia, are slow growing vascular lesions that present as one or more nodules in the soft tissues. It can also occur within osseous structures. It is most common in patients in their third through sixth decade of life with a mean age of onset in the early 30s. These lesions most commonly occur in the head and neck, most commonly in the periauricular region [19, 20]. These usually present as small erythematous dermal nodules in the head and neck, bleed easily and can itch [21]. Scarce literature is found on imaging characteristics of epithelioid hemangiomas, most reported as case reports. Soft tissue lesions have been described as well-circumscribed periorbital masses or as diffuse thickening and/or enlargement of the lacrimal gland [22]. Osseous lesions tend to present as well-defined expanding lytic lesions with thinned or destroyed cortex. MRI lesions have been described in literature as T1 hypointense, intermediate signal intensity on T2 with variable enhancement [23] (Fig. 3). Imaging plays a key role in determining extent especially when extensive reconstruction is expected as these lesions have been shown to involve the orbit, airway, and external auditory canal [24, 25].

Pyogenic granulomas, also known as lobular capillary hemangiomas, are relatively common and commonly present as a angiomatous pedunculated polyp or papule. These lesions commonly occur on the forehead and check, but can also occur on the mucosal surface including the conjunctiva and oral mucosa. These lesions most often occur secondary to prior trauma, infection, burns, and even pregnancy [26–28]. These lesions are usually first found on clinical examination often presenting as enlarging lesions over the course of a few weeks often with bleeding and ulceration. The underlying pathogenesis of these lesions is still unclear; historically, these were thought to be reactive hyperplasia. However, recent genetic studies showing mutations in *BRAF*, RAS, and GNA14 suggest that this is truly a benign neoplastic process. Sonographic evaluation usually demonstrates hypoechoic nodules in the region of interest with marked internal vascularity [29]. On MRI, these lesions present as T1 iso-intense to muscle with variable T2 signal. As expected, these lesions demonstrate avid enhancement [30]. Imaging can be used to guide biopsy and excision in more extensive cases.

### Locally aggressive/borderline

#### Kaposiform hemangioendothelioma

**Fig. 4:**
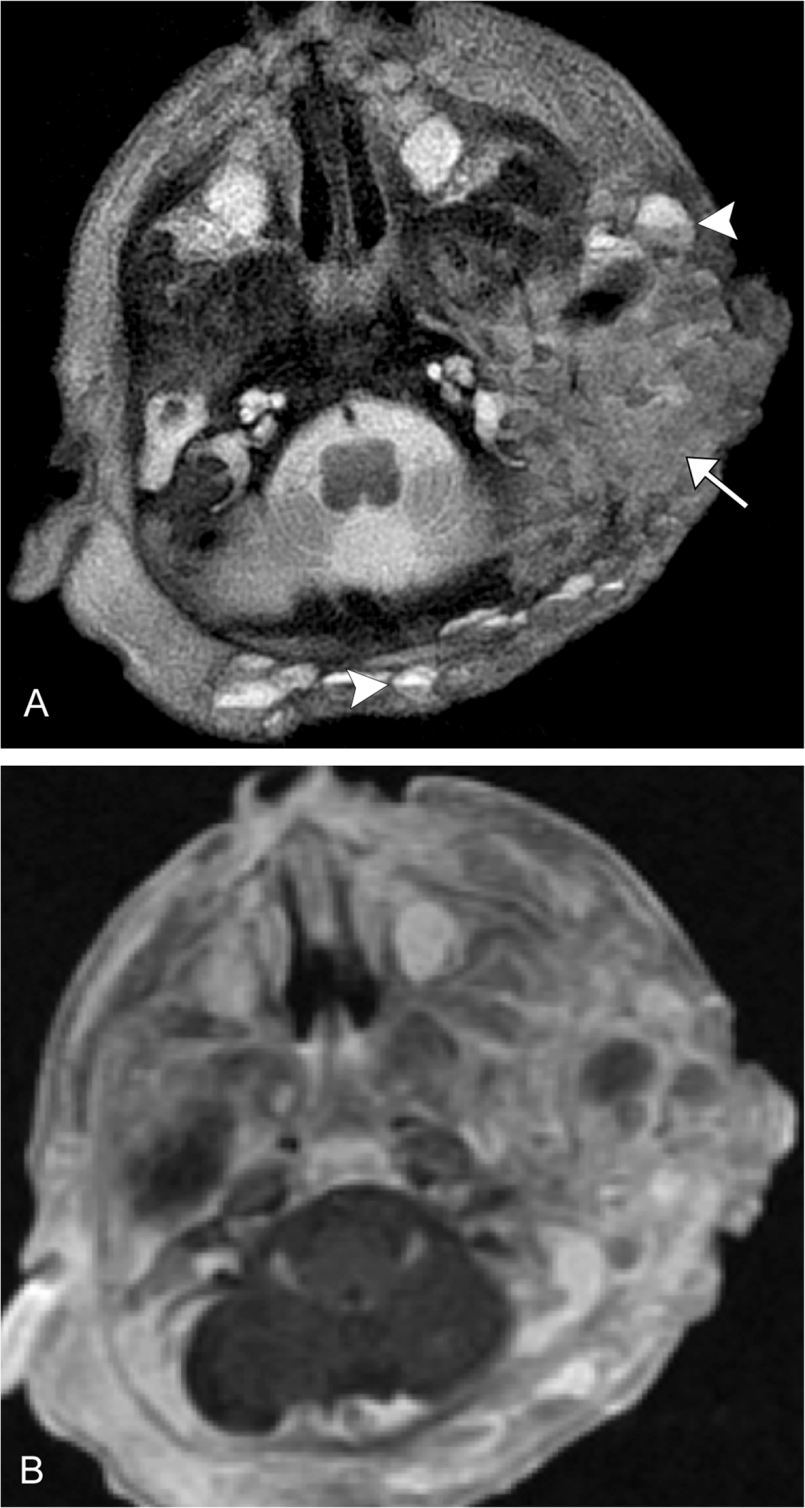
Kaposiform hemangioendothelioma. A 2-week-old premature male with large left neck mass. **a** Axial T2 image demonstrates an infiltrative, transpatial left facial heterogeneous mass that is predominantly mildly T2 hyperintense (arrow), and has multiple fluid-fluid levels (arrowheads). **b** Axial post-contrast fat-suppressed image demonstrates heterogeneous enhancement

Kaposiform hemangioendotheliomas are the most common aggressive vascular tumors, but still rare with a reported incidence between .07 and .091 per 100,000 children, presenting usually within the first year. It is an enlarging cutaneous lesion often associated with thrombocytopenia and pain. The majority of these lesions occur in the extremities, but about one-fifth occur in the head and neck region. On imaging, it is an ill-defined region of soft tissue thickening that crosses multiple planes. On T2-weighted imaging, the lesions demonstrate a hyperintense mass with an ill-defined margin that crosses multiple soft tissue planes. Additionally, there can be stranding of the subcutaneous fat, hemosiderin deposits, and destructive changes in the adjacent bone [31] (Fig. 4). This lesion is associated with GNA14, thought to act via a MAPK upregulation [32]. It is usually treated with propranolol and corticosteroids along with chemotherapeutic agents, classically vincristine. More recently, there has been good clinical success with mTOR inhibitors such as everolimus and sirolimus [33–35].

In addition to these, there are several other less common hemangioendotheliomas. These mainly affect adults. Overall, these commonly present as a solitary or several nodules in the skin, but can be seen elsewhere. They generally have a low metastatic potential, but can and often recur locally. Retiform hemangioendothelioma is a rare tumor with few cases and only one noted on the face. It has been shown to present as a dermal or subcutaneous nodule with slow growth over the course of months to several years. It is most common among young and middle-aged adults [36, 37]. These are similar to pseudomyogenic hemangioendothelioma [38]. Polymorphous hemangioendothelioma are extremely rare and characterized by varying patterns in different tumor areas. This is similar to composite hemangioendothelioma, which are actually composites of other types of hemangiomas. This variant can also include parts that resemble angiosarcoma [39].

Papillary intralymphatic angioendothelioma (PILA), also known as Dabska Tumor, is a rare locally aggressive tumor that involves the skin and subcutaneous tissues, with few case reports reporting lesions in the bone and other organs. It rarely metastasizes. On imaging, it has been shown to demonstrate aggressive features with isointense T1, heterogeneously increased T2 signal, and variable enhancement that can invade adjacent structures and demonstrate infiltrative characteristics [40, 41].

Lastly, there is Kaposi sarcoma (KS), a mesenchymal tumor that arises from lymphatic endothelial cells first described in 1872 [42]. KS garnered attention in literature and worldwide in the 1980s for its association with acquired immune deficiency syndrome (AIDS) [43]. Kaposi sarcoma herpesvirus/human herpes virus 8 (KSHV/HHV8) was identified in 1994 and plays a key role in the pathogenesis of KS, particularly in the setting of immune dysregulation [44, 45].

KS is divided into four variants: classic, endemic, iatrogenic (most commonly post-transplant), and AIDs related. Classic and endemic KS have a more indolent course and typically do not require imaging evaluation. Iatrogenic and AIDS-related KS are much more aggressive and often present with disseminated disease [43]. There has been significant decrease in AIDS-related cases since the introduction of highly active antiretroviral therapy (HAART) [46].

Conversely, due to increase number of transplants, biologic treatment, and long-term immunosuppression, the iatrogenic variant has been increasing in incidence [47, 48]. Patients typically present with multiple characteristic purple macules and papules, which can coalesce to form large plaques and tumors on the skin and can involve multiple organs. Cutaneous involvement is the most common presentation in head and neck KS seen in 66% of cases, 56% involve the mucosa, most commonly intraoral and laryngeal-pharyngeal mucosa, with the remainder presenting with isolated lymphadenopathy without papules. KS can be multifocal, which helps to differentiate it from other lesions [45, 49, 50]. CT and MR characteristics of KS are multiple nodular or polypoid lesions in the skin or mucosa. These lesions can vary in size and can infiltrate deep tissue however do not typically ulcerate. They most commonly demonstrate avid, enhancement on post-contrast imaging, however, can present as subtle asymmetries in the valleculae and pyriforum sinus [51]. In cases that present as isolated lymphadenopathy, it is important to have a good clinical history to help early diagnosis.

### Malignant

#### Angiosarcoma

**Fig. 5:**
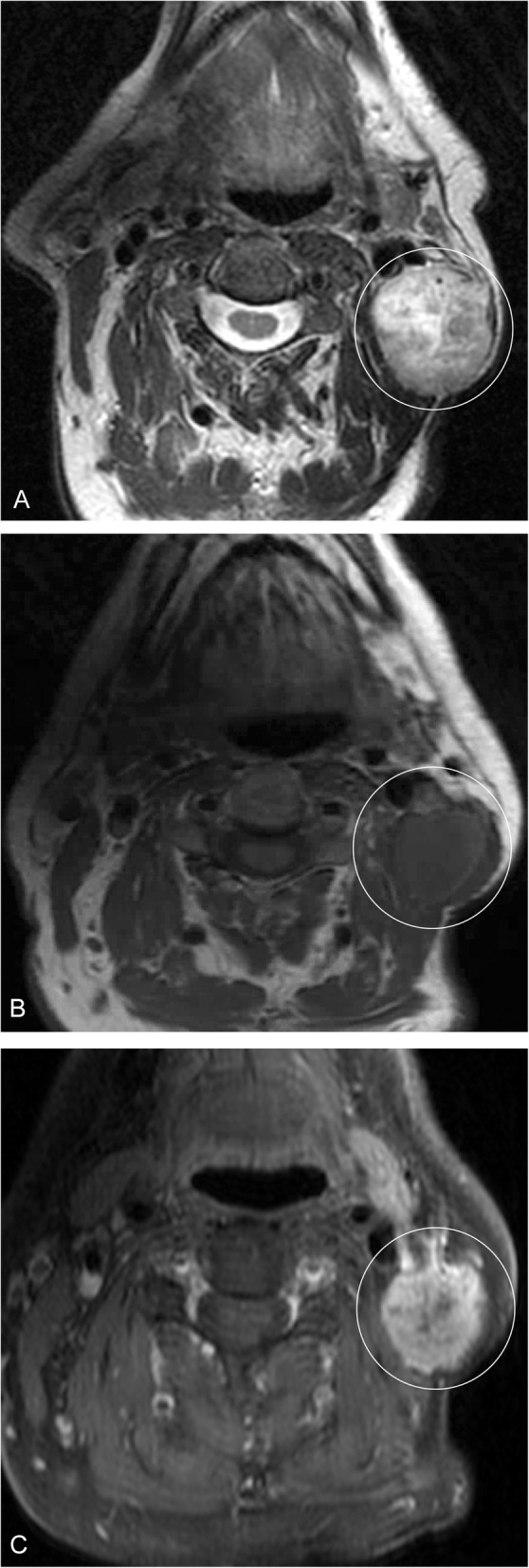
Angiosarcoma. A 54-year-old male presents with an enlarging left neck mass. **a** Axial T2 image demonstrates a heterogeneous, mostly hyperintense mass in the left neck (circled). **b** T1 pre- and (**c**) post-contrast fat-suppressed images demonstrate heterogeneous enhancement of the lesion (circled). These imaging features are non-specific; the patient had biopsy of the lesion, which was pathology proven angiosarcoma

Angiosarcoma is a rare aggressive soft tissue malignancy arising from endothelial cells. Overall survival is approximately 6 to 16 months as these tumors have a propensity to recur and metastasize [52]. Angiosarcoma can occur at any age with the reported median age between 60 and 71 years [53]. There is a male predilection for soft tissue angiosarcomas; however, the incidence of deep soft tissue and parenchymal tumors is the same in men and women [52, 54]. Approximately 50% of angiosarcomas occur in the head and neck soft tissue, particularly the scalp; 10% occur in the deep soft tissue including the peritoneum, retroperitoneum, and mediastinum; and the remainder present in parenchymal organs including breast, bone, and liver. Lung and bone are the most common site for metastasis through hematogenous spread. The two most common risk factors are chronic lymphedema and radiation therapy [55]. Patients often present with skin lesions such as an enlarging bruise, a discolored nodule, or persistent ulceration. In the early stages, these lesions can be misdiagnosed for benign entities caused by cellulitis, infection, or skin injuries [56]. Typical imaging characteristics include intermediate T1 signal intensity and high T2 heterogeneity with avid enhancement. Flow voids or high-flow serpentine loss of signal on T1 and T2 imaging in a soft tissue mass is characteristic of the lesions [57]. On contrast-enhanced CT, lesions are typically irregular, enhancing soft-tissue masses with possible invasion of adjacent osseous and parenchymal structures [56] (Fig. 5).

#### Epithelioid hemangioendothelioma

**Fig. 6:**
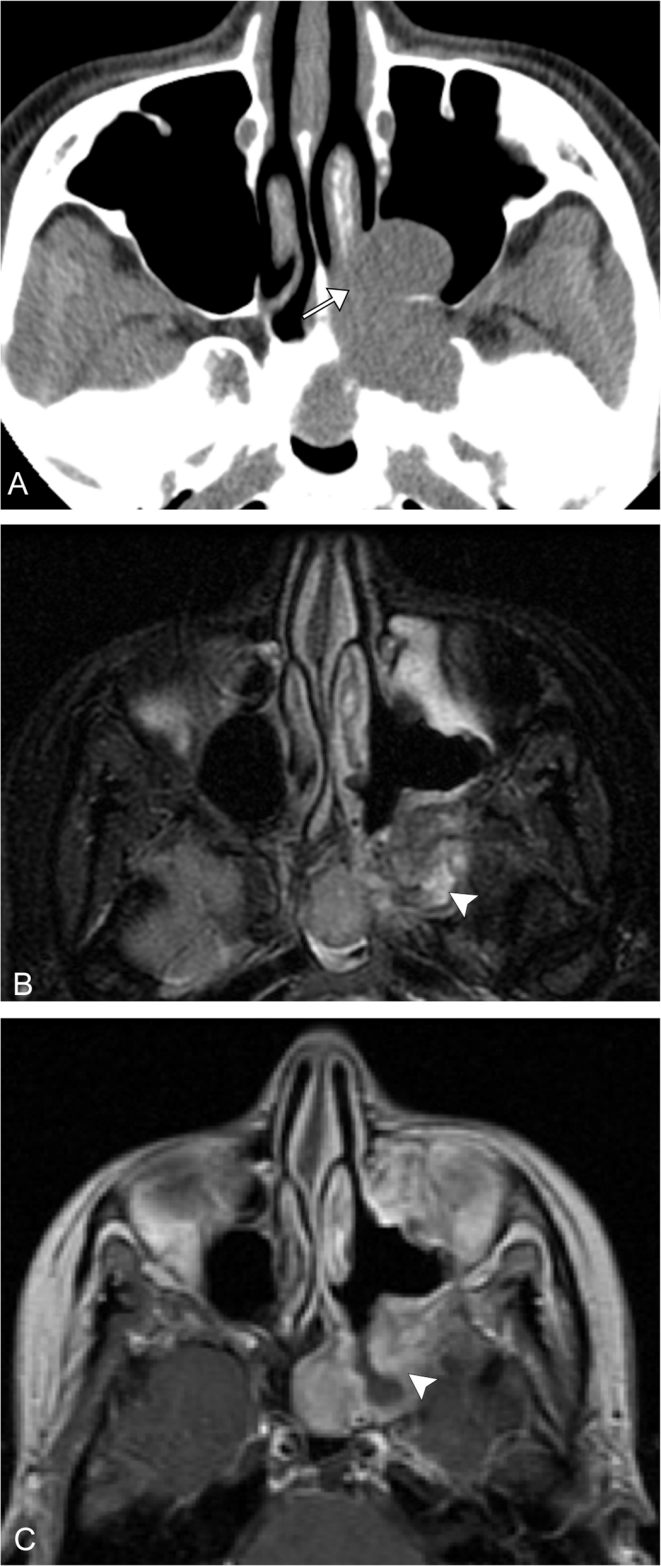
Epithelioid hemangioendothelioma. A 37-year-old female with left facial fullness and recurrent sinusitis. **a** Axial CT image shows an expansile polypoid lesion centered in the left pterygopalatine fossa, extending into the maxillary sinus and sphenoid sinus, as well as the left nasal cavity (arrow). **b** Axial T2 image after diagnostic and therapeutic resection demonstrates residual ill-defined mildly T2 hyperintense tissue along the peripheral margins of resection (arrowhead). **c** Corresponding axial T1 post-contrast image demonstrates avid enhancement of the residual lesion (arrowhead)

Epithelioid hemangioendothelioma (EHE) is a rare vascular tumor first described in 1975 as an aggressive bronchoalveolar cell carcinoma [58]. Later named EHE by Weiss and Enzinger in 1982 for its similar features between a hemangioma and angiosarcoma [58]. EHE is exceedingly rare and accounts for 1% of all vascular malignancies; it is often misdiagnosed at presentation. Most commonly it affects the lungs, liver, or bones, although can present in the head and neck area, mediastinum, among many other sites. EHE is often incidentally diagnosed and over 50–76% of patients are asymptomatic. Typically, the lesion presents in patients between the age of 30 and 50 years [59, 60]. There is limited literature describing imaging characteristics of EHE involving the head and neck, but aggressive features can suggest the diagnosis (Fig. 6).

#### Others

The lesions discussed above are not inclusive of all vascular malignant vascular tumors; there are others, which are exceedingly rare and mostly differentiated on pathology.

## Vascular malformations

**Fig. 7:**
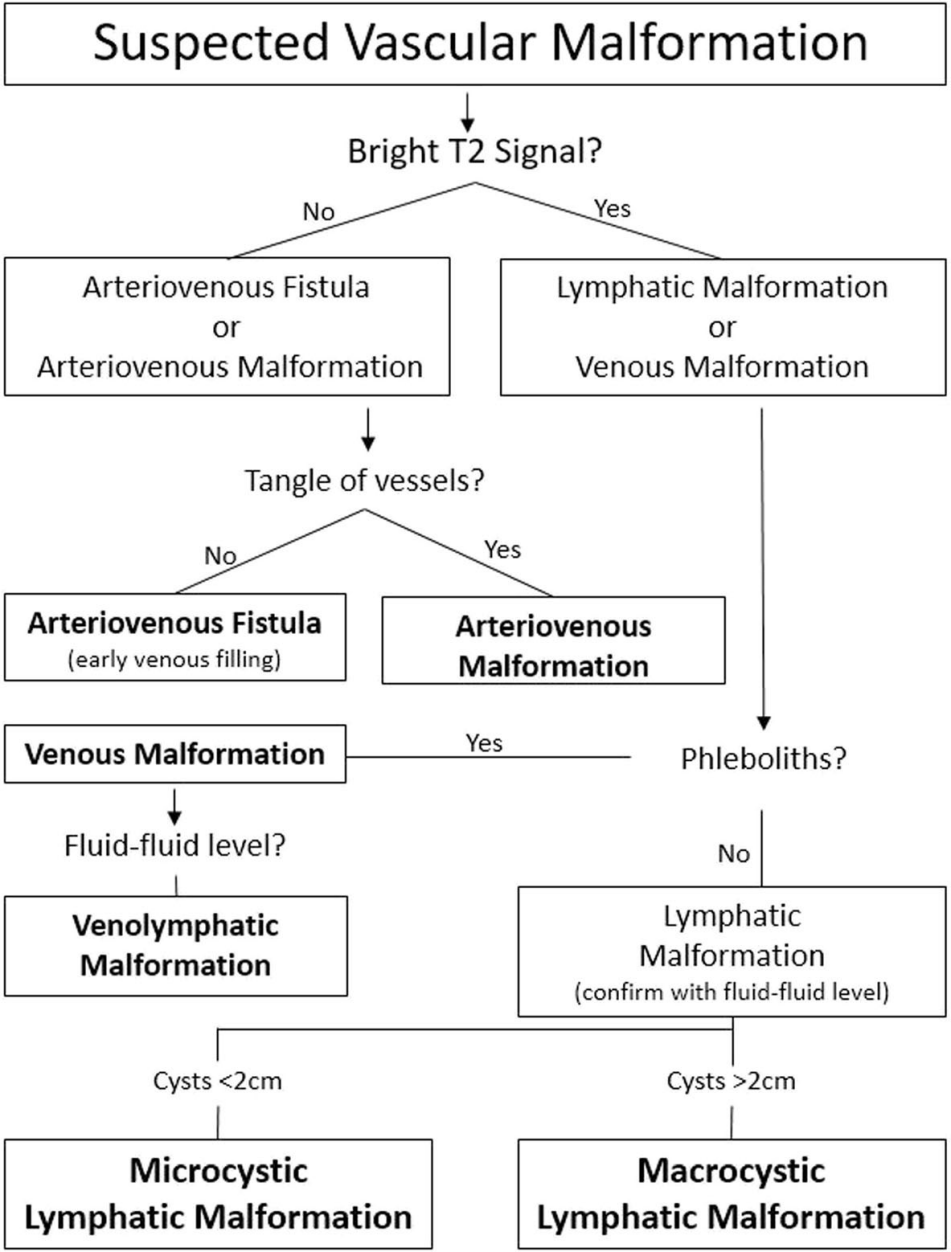
Imaging-based algorithm for suspected vascular malformations

Vascular malformations make up the second set of anomalies and are broken up into four categories. Simple malformations include capillary, lymphatic, venous, arteriovenous malformations, and arteriovenous fistulas. These are usually described on imaging based on flow rates through the lesion, slow or fast. Combined malformations include lesions with more than one type of malformation, such as capillary-venous, lymphatic-venous, etc. Malformations of major vessels and those associated with other anomalies make up the last two categories (Fig. 7).

### Capillary malformations

Capillary malformations are cutaneous lesions that often follow a dermal pattern. The diagnosis is usually clinical as a “port wine stain” is usually noted on routine physical exam. On ultrasound, these lesions are isoechoic and confined to the dermis; Doppler flow can be seen occasionally. The main utility of imaging is in the setting of associated findings (i.e., Sturge-Webber). The mainstay of treatment is laser therapy, but surgical excision with reconstruction can also be considered [61].

### Venous

**Fig. 8:**
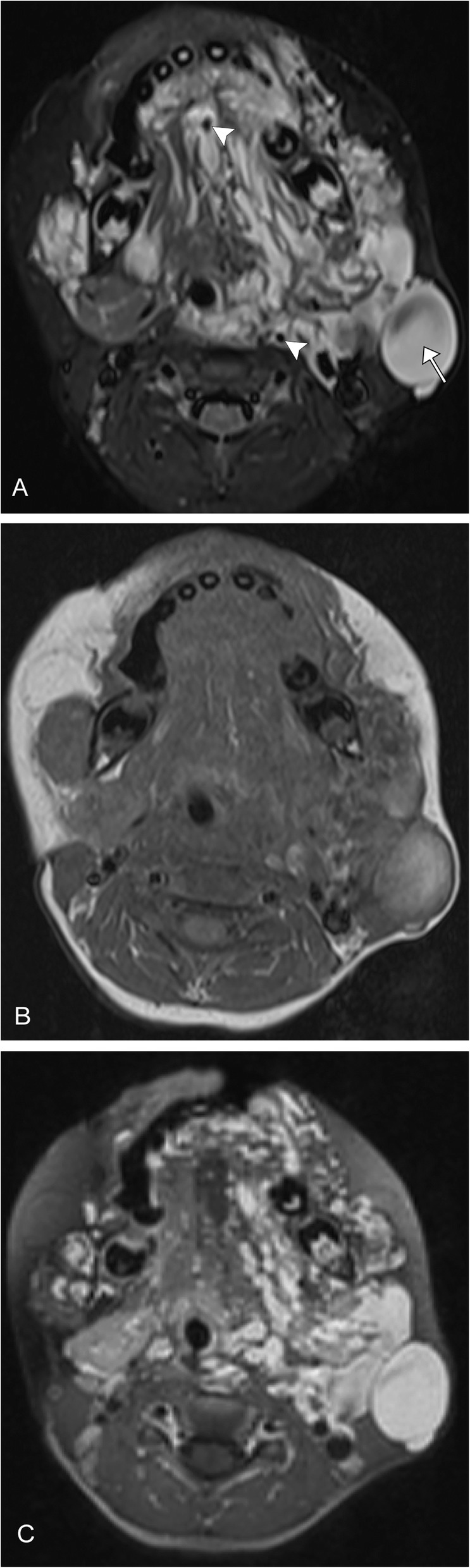
Venous malformation. A 3-year-old female with left neck fullness and overlying bluish-purple discoloration. **a** Axial T2 fat-saturated image demonstrates tortuous high signal lesions involving predominantly the left neck and face (arrow). Several scattered internal foci of low T2 signal are consistent with phleboliths (arrowheads), pathognomonic for this lesion. **b** Axial T1 pre-contrast image shows the lesion to be isointense to adjacent muscle. **c** Axial T1 post-contrast image demonstrates avid enhancement of the tubular structures

Venous malformations make up most slow flow lesions. These lesions initially present during mid to late childhood. They are continuous, soft, and compressible lesions that involve several layers. They tend to align with muscle groups or track along nerves or major vessels. They are responsive to changes in venous flow such as Valsalva or sometimes compression of the ipsilateral jugular vein when the lesion is in the head and neck. On ultrasound, these lesions appear as multiple tubules and may or may not have detectible flow due to low velocities. MRI is the mainstay for evaluating venous malformations. These lesions are markedly T2 hyperintense with serpiginous tubules. One of the most common and highly specific differentiating features is the presence of phleboliths. These lesions can thrombose [10, 61]. CT is best for the evaluation of the presence of phleboliths, as well as in cases of osseous involvement (Fig. 8).

### Lymphatic

**Fig. 9:**
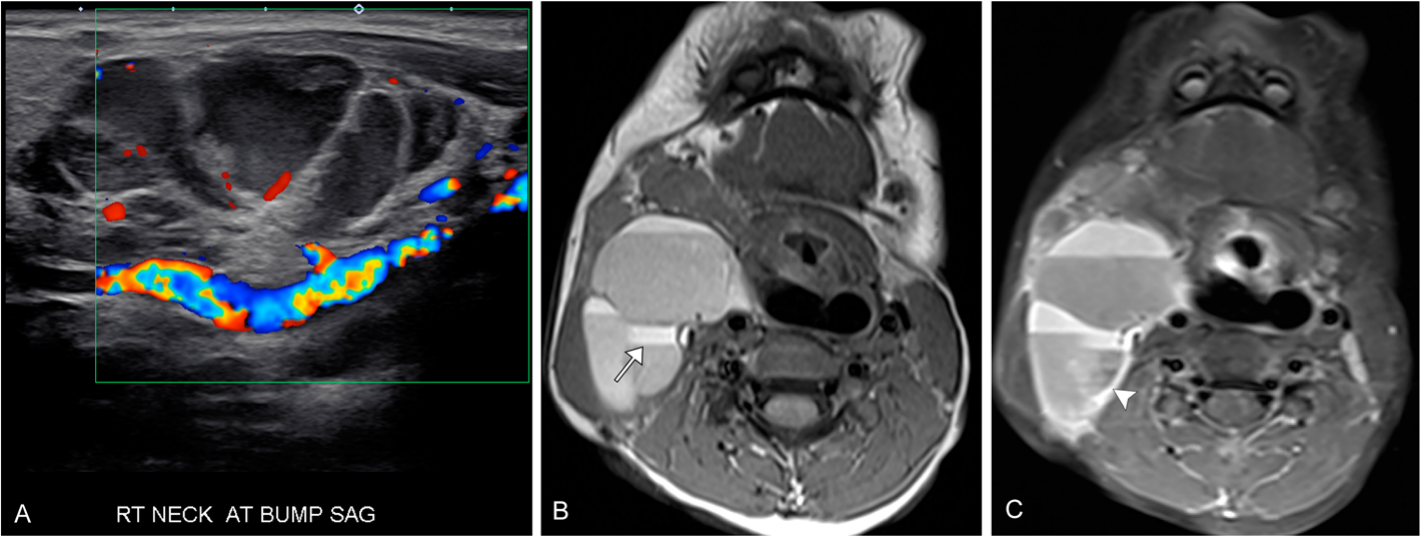
Macrocystic lymphatic malformation. A 2-year-old male with right neck mass. **a** US demonstrates a complex multiloculated cystic mass without internal vascularity. **b** Axial T1 pre-contrast image better demonstrates the multiloculated cystic mass, which has fluid-fluid levels (arrow). **c** Axial T1 post-contrast fat-saturated image demonstrates enhancement of the walls and septa (arrowhead). However, there is no internal enhancement within the lesion

**Fig. 10:**
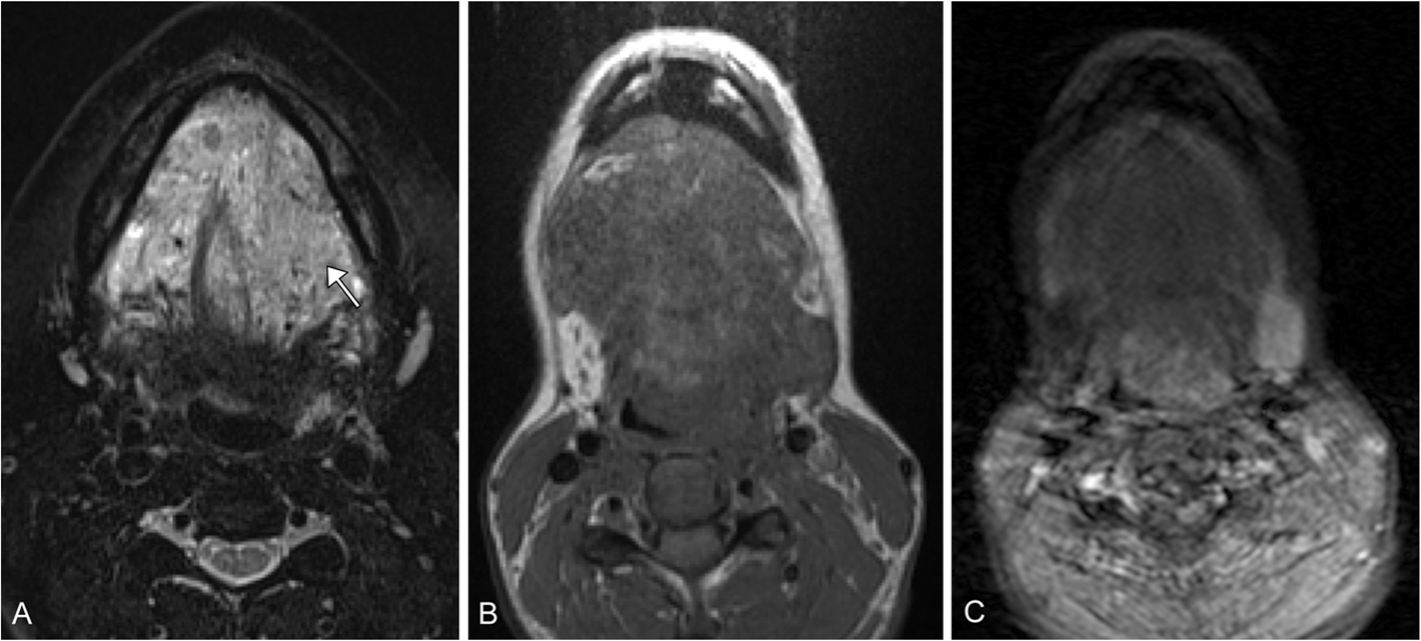
Microcystic lymphatic malformation. An 11-year-old female with progressive mouth fullness. **a** Axial T2 fat-saturated image demonstrates a transpatial hyperintense lesion within the floor of the mouth (arrow) containing tiny cysts. **b** Axial T1 image demonstrates the lesion to be hypointense. **c** Axial T1 post-contrast image demonstrates no enhancement

Lymphatic malformations are overall very rare, but the majority, approximately three-fourth, occur in the head and neck region. One review found the bulk of these occur in the cheek, around the mandible and parotid gland. They are soft and compressible cystic structures, which usually present within the first two years of life. These cystic structures can be macrocytic (greater than 2 cm) or microcystic (less than 2 cm). Mixed lesions with macro- and microcystic components can also occur. On ultrasound, they appear as cystic structures without flow. On MRI, they usually have high T2 signal with minimal peripheral enhancement. The T1 signal is variable. These lesions have a tendency to hemorrhage, and therefore fluid-fluid levels are key to the correct diagnosis (Fig. 9). An important variation is a diffuse microcystic lymphatic malformation, as these may present with mild diffuse enhancement of the cyst walls (Fig. 10). These lesions can be difficult to treat as both surgery and sclerotherapy have high rates of recurrence, with the possibility of aggravation [10, 62].

### High flow lesions

**Fig. 11:**
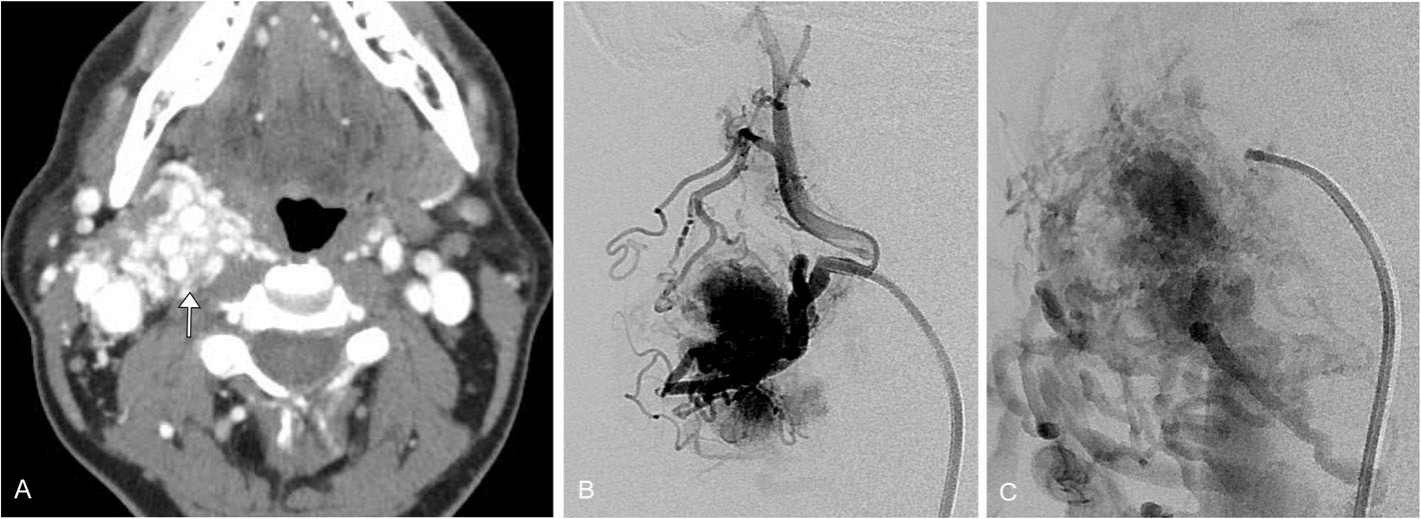
Arteriovenous vascular malformation. A 50-year-old male with right neck fullness. **a** Contrast-enhanced axial CT shows a tangle of serpiginous vessels in the right deep neck (arrow). **b** Conventional angiogram with injection of an external carotid artery dominant feeder shows brisk enhancement of the nidus. **c** Delayed angiographic image shows multiple draining venous vessels

**Fig. 12:**
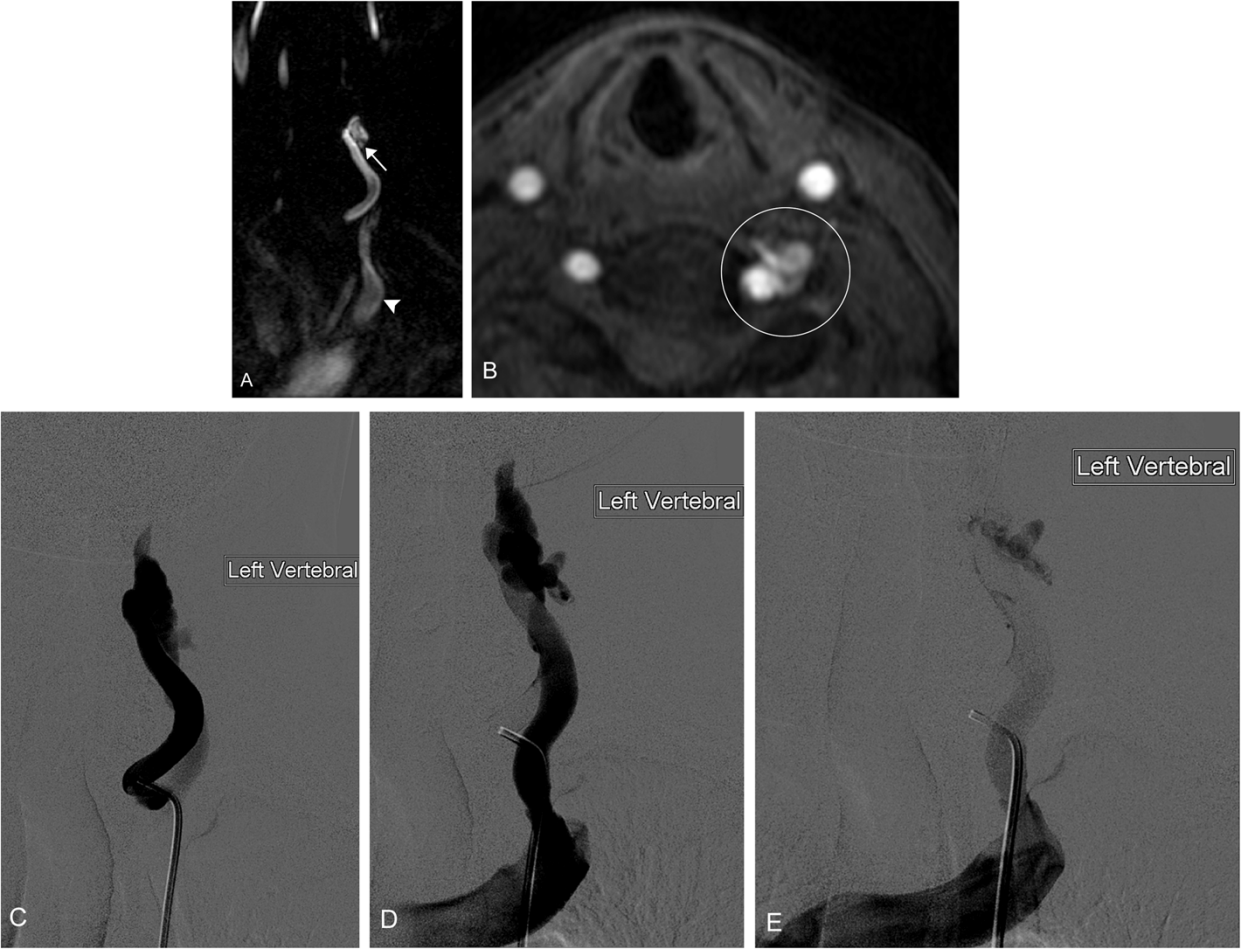
Arteriovenous fistula. A 57-year-old female presents with history of left neck bruit. **a** Post-contrast MRA images of the neck demonstrate a tortuous course of the left vertebral artery extending into fistulous enlargement at the C6 level (arrow) where there is early filling into the draining vein (arrowhead). **b** Axial post-contrast MRA image demonstrates fistulous enlargement of the vertebral artery (circle). **c** Conventional angiography with selective injection of the left vertebral artery shows enlargement at the proximal portion. **d, e** Progressive angiographic images show early and abnormal filling of the large draining vein, ultimately draining into the brachiocephalic vein

Arteriovenous malformations and fistulae are high flow anomalies. These malformations lack the normal capillary bed between vessels. They can be congenital or occur secondary to surgery, trauma, and pregnancy. They may present with a skin deformity, pulsatile mass, bleeding, ulceration, or secondary symptoms including arterial steal, venous congestion, and high output cardiac failure. These lesions demonstrate high flow on ultrasound; it can be difficult to identify the arterial origin or venous drainage of these lesions. CT is employed to evaluate bony involvement. MRI can be used to evaluate soft tissue involvement. Key to diagnosis is that these lesions show flow voids on both T1W and T2W sequences. Conventional arteriography can be both diagnostic and therapeutic if there is embolization of the lesion (Figs. 11 and 12). Other options for treatment include surgical excision and radiation [2, 10, 61].

### Combined malformations

**Fig. 13:**
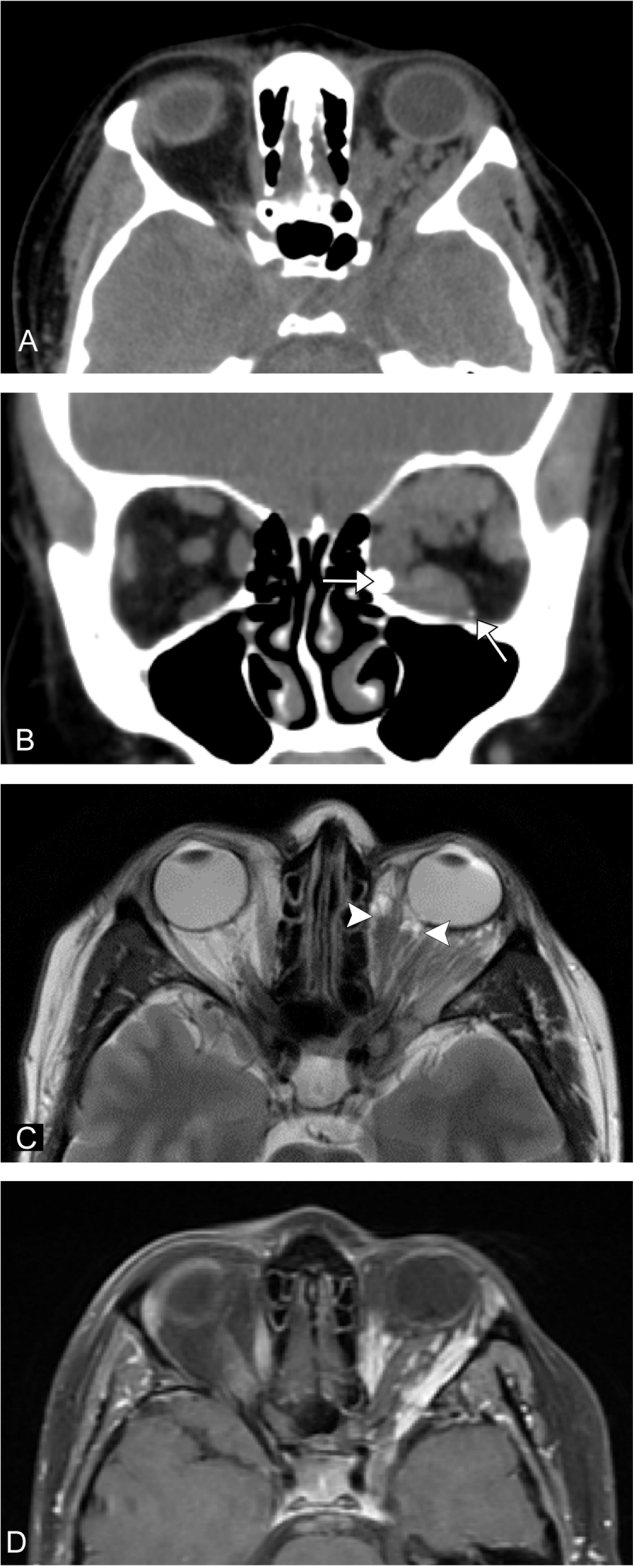
Venolymphatic malformation. A 31-year-old female presents with left eye fullness. **a** Axial CT image demonstrates a serpiginous lesion within the left orbit causing proptosis. **b** Coronal CT image demonstrates a few phleboliths at the inferomedial margin (arrows). **c** Axial T2-weighted image shows fluid-fluid levels within the lesion (arrowheads). **d** Axial T1 post-contrast fat-saturated image shows enhancement of portions of the lesion

These lesions are complex and include more than one type of malformation. These lesions are highly variable and can present unique challenges in both diagnosis and treatment. It is important to determine if the lesion is high or slow flow or a combination. These lesions can be evaluated by MRI and magnetic resonance venography, which can help delineate between venous and lymphatic components (Fig. 13). Additionally conventional venography or arteriography with varying techniques including direct puncture, tourniquets, and other maneuvers can be used to delineate components of the malformation. It is important to rule out collateral involvement of major neck and intracranial vessels. A multidisciplinary approach is essential to treating these lesions [63, 64].

### Associations with other anomalies

There are numerous malformations associated with syndromes. In this review, we have included multiple related to the head and neck. The most commonly seen is Sturge-Weber. These patients have facial capillary malformations with variable involvement of the leptomeninges and orbits [65]. This anomaly is tied to a mutation in GNAQ, which impacts cell differentiation, proliferation, and inhibits apoptosis but is not oncogenic in this setting [66].

Congenital, lipomatous overgrowth, vascular malformations, epidermal nevi, and skeletal/spinal abnormalities (CLAPO) syndrome is characterized by capillary malformations of the lower lip often with lymphatic and venous malformations of the head and neck. Additionally, these patients have variable degrees of asymmetric overgrowth. It is important to look for venous and lymphatic malformations in patients with suspected CLAPO syndrome. Other common findings include facial infiltrating lipomatosis. Interestingly, these patients do not have neurological findings seen in other similar syndromes, likely due to the embryonic origin of the involved tissues [67, 68].

Congenital, lipomatous, overgrowth, vascular malformations, epidermal nevi and spinal/skeletal anomalies and/or scoliosis (CLOVES) syndrome is associated with several kinds of malformations, including lymphatic, venous, capillary, and even arteriovenous malformations, along with lipomatous overgrowth. Patients with CLOVES syndrome often have cranial abnormalities including megalocephaly, Chiari malformation, and cortical dysplasia, among others. These findings should be considered when evaluating CLOVES syndrome patients [69, 70]. Several other malformations are associated with gross cephalic abnormalities. Both macrocephaly and microcephaly are associated with capillary malformations. Bannayan-Riley-Ruvalcaba syndrome is characterized by macrocephaly, as well as AVMs and VMs [71]. Lastly, patients with Proteus syndrome present with dolichocephalic, hyperostosis, and asymmetric overgrowth; they can have capillary, venous, or lymphatic malformations with asymmetrical somatic overgrowth [72]. As a whole, these are rare syndromes with a significant amount of variability; however, if several malformations are seen, one should consider one of these syndromes and tailor further studies accordingly.

## Conclusion

It is important to remember that vascular anomalies of the head and neck can be divided into vascular tumors and vascular malformations; the former representing true proliferative neoplasms and the latter defects of vascular morphogenesis. These lesions can present in a variety of locations in the head and neck. Imaging plays a role for many of these lesions, and therefore, knowledge of the classification of these lesions based on the updated 2018 International Society for the Study of Vascular Anomalies, as well as characteristic imaging findings are key to diagnosis and subsequent appropriate treatment.

## Data Availability

Not applicable.

## Abbreviations

AIDS: Acquired immune deficiency syndrome
CLAPO: Congenital, Lipomatous overgrowth, vascular malformations, epidermal nevi, and skeletal/spinal abnormalities
CLOVES: Congenital, lipomatous, overgrowth, vascular malformations, epidermal mevi and spinal/skeletal anomalies and/or scoliosis
EHE: Epithelioid hemangioendothelioma
GLUT1: Glucose transporter protein-1
HAART: Highly active antiretroviral therapy
ISSVA: International Society for the Study of Vascular Anomalies
KHE: Kaposiform hemangioendothelioma
KS: Kaposi sarcoma
KSHV/HHV8: Kaposi sarcoma herpesvirus/human herpes virus 8
MRI: Magnetic resonance imaging
NICH: Non-involuting capillary hemangioma
PHACE syndrome: Posterior fossa malformations, hemangiomas, arterial anomalies, cardiac abnormalities, eye anomalies
PICH: Partially involuting congenital hemangiomas
PILA: Papillary intralymphatic angioendothelioma
RICH: Rapidly involuting congenital hemangiomas
